# Supplementary material for: A neural circuit linking learning and sleep in Drosophila long-term memory
Source: Nat Commun. 2022 Feb 1;13:609. doi: 10.1038/s41467-022-28256-1 (PMC8807839; doi:10.1038/s41467-022-28256-1)
Supplement: Supplementary file 1 — Supplementary Information [file 41467_2022_28256_MOESM1_ESM.pdf]

Supplementary information

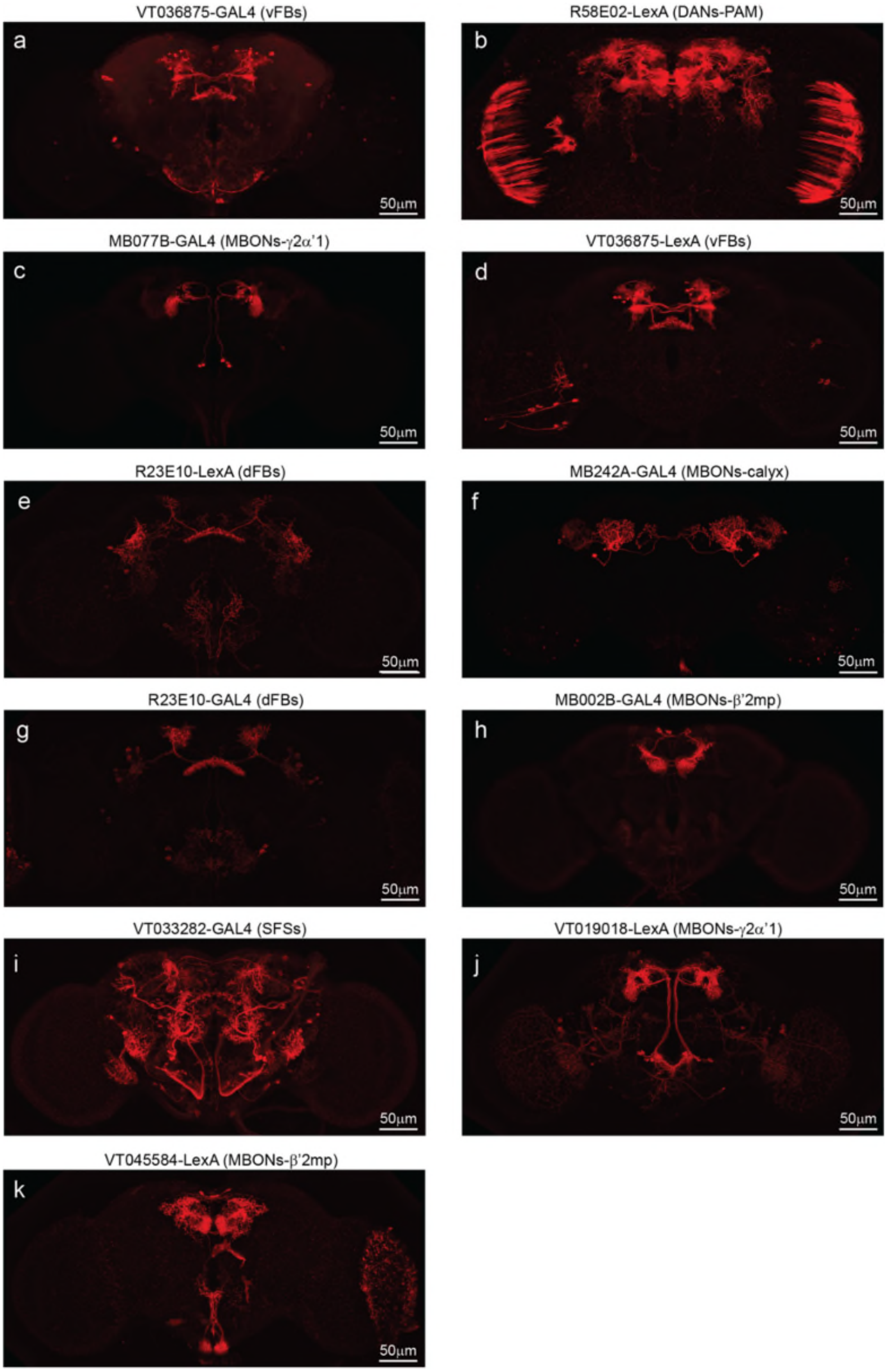

Supplementary Figure. 1 | Expression pattern of the driver lines used in this study. Confocal images of central brains carrying indicated driver lines and either *UAS-myrGFP* or *LexAop-myrGFP* tag stained with corresponding antibodies to reveal neural projections. Scale bar = 50µm. The expression pattern images of selected lines can be viewed at https://flweb.janelia.org/cgi-bin/flew.cgi and https://gen1mcfo.janelia.org/cgi-bin/gen1mcfo.cgi. n = 5 fly samples per line.

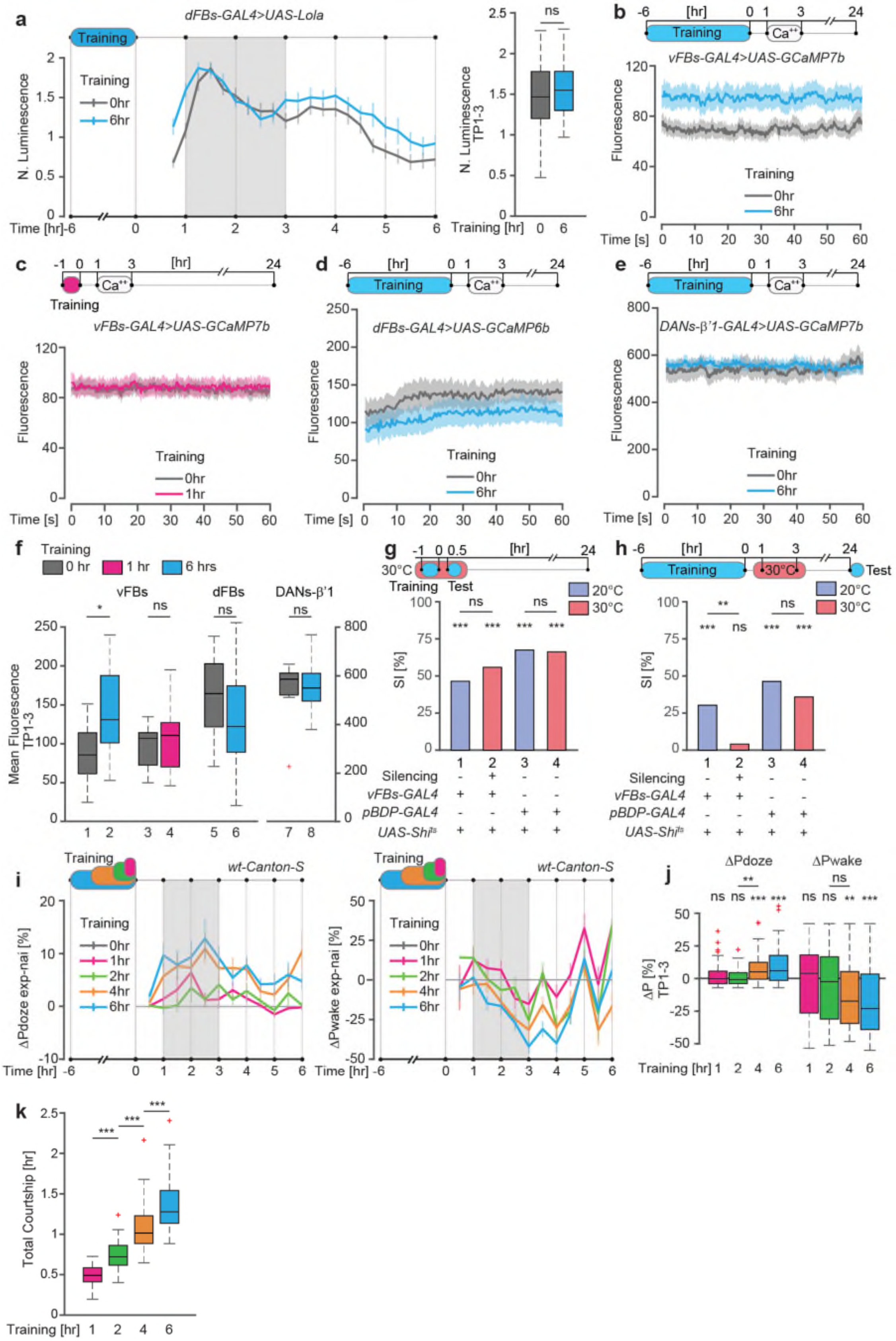
a
dFBs-GAL4>UAS-Lola
Training
N. Luminescence
Training
0hr
6hr
Time [hr]
N. Luminescence TP1-3
ns
Training [hr]
b
vFBs-GAL4>UAS-GCaMP7b
Training
Ca++
Fluorescence
Training
0hr
6hr
Time [s]
c
Training
vFBs-GAL4>UAS-GCaMP7b
Fluorescence
0hr
1hr
d
dFBs-GAL4>UAS-GCaMP6b
e
DANs-β'1-GAL4>UAS-GCaMP7b
f
Training
0 hr
1 hr
6 hrs
vFBs
dFBs
DANs-β'1
Mean Fluorescence TP1-3
g
Training
Test
20°C
30°C
SI [%]
Silencing
vFBs-GAL4
pBDP-GAL4
UAS-Shi^ts
h
Test
i
wt-Canton-S
ΔPdoze exp-nai [%]
ΔPwake exp-nai [%]
Training
0hr
1hr
2hr
4hr
6hr
j
ΔPdoze
ΔPwake
ΔP [%] TP1-3
Training [hr]
k
Total Courtship [hr]
Training [hr]

Supplementary Figure 2 | vFBs are activated by a learning experience that induces LTM and post-learning sleep. a. (left) Normalized mean luminescence traces (+/- SEM) of dFB neurons. (right) Mean normalized luminescence in the 1-3 hour time period (TP1-3) after training on the left. n = 40 and 34 flies for naïve and trained group respectively, P = 0.4339 for H_0 N. $Lum._{exp}$ = N. $Lum._{nai}$, two-sided Student T-test. **b**, **c**. Mean calcium level traces (+/- SEM) in vFBs measured for 1 min at multiple time points spanning TP1-3. **d, e.** Mean calcium level traces (+/- SEM) in dFBs and DANs-β'1 measured for 1 min at multiple time points spanning the TP1-3. **f.** Box plot of the data in b, c, d, e. n_1 = 27, n_2 = 2, n_3 = 20, n_4 = 16, n_5 = 11, n_6 = 14, n_7 = 17, n_8 = 10, P_{12} = 0.0431, P_{34} = 0.7583, P_{56} = 0.1948, P_{78} = 0.8179 for H_0 F_{exp} = F_{nai}, two-sided Student T-test. **g.** Short-term memory **(**STM) represented as SI [%], upon silencing of vFBs. n_1 = 35 and 35, n_2 = 41 and 42, n_3 = 41 and 40, n_4 = 39 and 38 for naïve and trained group respectively, P_1 = 1.0e-5, P_2 = 1.1e-4, P_3 = 1.0e-5, P_4 = 1.0e-5 for H_0 SI = 0, and P_{12} = 0.4951, P_{34} = 0.9021 for H_0 SI_{exp} = SI_{ctrl}, two-sided Permutation test. **h.** Long-term memory (LTM) represented as SI [%], upon SFSs silencing. n_1 = 69 and 61, n_2 = 71 and 69, n_3 = 71 and 64, n_4 = 69 and 65 for naïve and trained group respectively, P_1 = 1.0e-5, P_2 = 0.1716, P_3 = 1.0e-5, P_4 = 1.0e-5 for H_0 SI = 0, and P_{12} = 0.0028, P_{34} = 0.2582 for H_0 SI_{exp} = SI_{ctrl}, two-sided Permutation test. **i.** (left) Mean change in probability (+/- SEM) of falling asleep (ΔP doze) or (right) waking up (ΔP wake). **j**. ΔP doze and ΔP wake in TP1-3 in **i**. n_{0hr} = 45, n_{1hr} = 42, n_{2hr} = 41, n_{4hr} = 44, n_{6hr} = 48, P_{1hr} = 0.3325, P_{2hr} = 0.6087, P_{4hr} = 0.0002, P_{6hr} = 9.3e-5 for H_0 ΔPdoze = 0; P_{1hr} = 0.8930, P_{2hr} = 0.5578, P_{4hr} = 0.0065, P_{6hr} = 5.4e-5 for H0 ΔPwake = 0, two-sided Wilcoxon Signed Rank test, P = 0.0049 for H_0 $ΔPdoze_{2hr}$ = $ΔPdoze_{4hr}$ and P = 0.1464 for H0 $ΔPwake_{2hr}$ = $ΔPwake_{4hr}$, two-sided Wilcoxon Rank Sum test. **k**. Cumulative courtship time (CT) upon increasing duration of training. n = 54 flies. P = 9.7e-12 for H_0 CT_{1hr} = CT_{2hr}, P = 5.8e-19 H_0 CT_{2hr} = CT_{4hr}, P = 3.4e-19 H_0 CT_{4hr} = CT_{6hr}, two-sided Wilcoxon Rank Sum test. Full genotypes and data analysis details in Supplementary Table S1, S2 and S3. Source data are provided as a Source Data file. ns P > 0.05, *P < 0.05, **P < 0.01, ***P < 0.001. n represents independent fly samples with assays repeated at least 3 times (**a**, **f**, **g**, **h**, **j** and **k)**. Box plots represent median and IQR, whiskers extend to lower and upper adjacent values and red crosses for outliers (**a**, **f**, **j** and **k**).

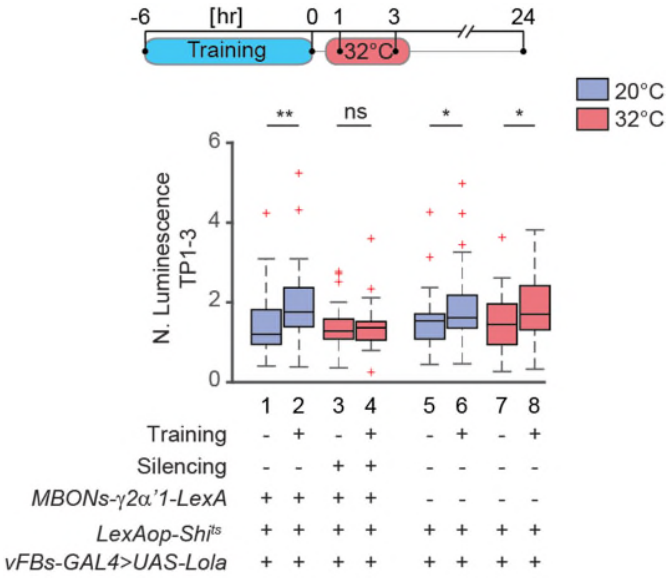

Supplementary Figure 3 | Activity of MBONs-γ2α'1 is essential for the increased vFBs activity after prolonged learning. Mean normalized luminescence of vFBs in the 1-3 hour time period (TP1-3) upon MBONs-γ2α'1 silencing. $n_1 = 51$, $n_2 = 51$, $n_3 = 39$, $n_4 = 42$, $n_5 = 42$, $n_6 = 47$, $n_7 = 43$, $n_8 = 44$, $P_{12} = 0.0062$, $P_{34} = 0.8902$, $P_{56} = 0.0470$, $P_{78} = 0.0217$ for H_0 $Lum._{exp} = Lum._{nai}$, ns $P > 0.05$, $*P < 0.05$, $**P < 0.01$, two-sided Student T-test. Full genotypes details in Supplementary Table S1 and S2. Source data are provided as a Source Data file. Box plots represent median and IQR, whiskers extend to lower and upper adjacent values and red crosses for outliers.

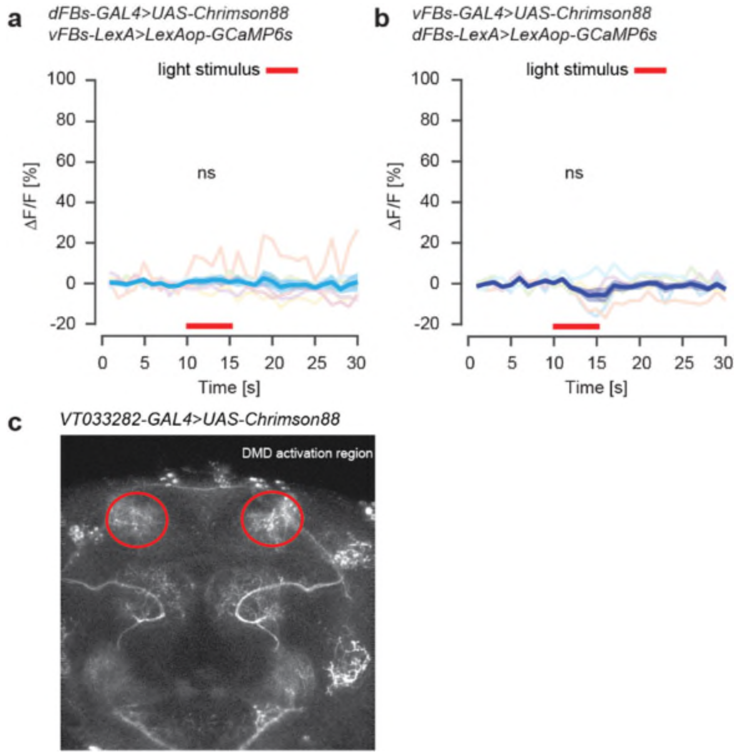

Supplementary Figure 4 | vFBs and dFBs are not functionally connected. a. Traces of mean GCaMP6s responses (ΔF/F) (+/- SEM) in vFBs upon optogenetic activation (red bar) of dFBs. n = 7 flies, $P = 0.4777$ for H_0 ΔF/F = 0, ns $P > 0.05$, two-sided Student T-test. **b.** Traces of mean GCaMP6s responses (ΔF/F) (+/- SEM) in dFBs upon optogenetic activation (red bar) of vFBs, n = 7 flies, $P = 0.1154$ for H_0 ΔF/F = 0, ns $P < 0.05$, Student T-test. **c.** Expression pattern of *VT033282>UAS-Chrimson88-tdTomato* with depicted region (red circles) for targeted photoactivation with the Digital Mirror Device (DMD). Full genotypes details in Supplementary Table S1, S2 and S3.

Supplementary Tables

Type	Short name	Full genotype
wild-type	*Canton S*	*Canton-S wild type*
reporter	*LexAop-pGCaMP6s*	*13xLexAop2-Syn21-opGCaMP6s in attp8*
reporter	*UAS-Lola*	*UAS-FLP.PEST; lola>>luc*
reporter	*UAS-GCaMP6s*	*20xUAS-Syn21-opGCaMP6s in attp8*
reporter	*UAS-GCaMP7b*	*20xUAS-IVS-Syn21-Syt::Op-jGCaMP7b in attP40*
reporter	*UAS-mGFP*	*10XUAS-IVS-mCD8::GFP in attP2*
effector	*LexAop-Chrimson88*	*13xLexAop2-Chrimson88-tdTomato in attp18*
effector	*LexAop-GAL80*	*LexAop-GAL80 in attp40*
effector	*UAS-Chrimson88*	*10xUAS-Chrimson88-tdTomato in attp18*
effector	*UAS-CsChrimson*	*20xUAS-CsChrimson-mVenus in attP18*
effector	*UAS-Shi^{ts}*	*UAS-Shibire^{ts} in attp40*
effector	*LexAop-Shi^{ts}*	*LexAop-Shibire^{ts} in vk00005*
driver	*MB002B*	ref [1]
driver	*MB018B*	ref [1]

driver	*MB027B*	ref [1]
driver	*MB051B*	ref [1]
driver	*MB052B*	ref [1]
driver	*MB057B*	ref [1]
driver	*MB077B*	ref [1]
driver	*MB082C*	ref [1]
driver	*MB083C*	ref [1]
driver	*MB110C*	ref [1]
driver	*MB112C*	ref [1]
driver	*MB242A*	ref [1]
driver	*MB310C*	ref [1]
driver	*MB399B*	ref [1]
driver	*MB433B*	ref [1]
driver	*MB434B*	ref [1]
driver	*MB542B*	ref [1]
driver	*MB543B*	ref [1]
driver	*MB549C*	ref [1]
driver	*MB622B*	ref [1]
driver	*pBDP-GAL4*	*pBDP-GAL4 in attp2*
driver	*R23E10-GAL4*	*R23E10-GAL4 in attp2* [2]
driver	*R23E10-LexA*	*R23E10-LexA4 in attp40* [2]
driver	*R58E02-LexA*	*R58E02-LexA in attp40* [2]
driver	*SS01308-GAL4*	*VT50240-p65ADZp in attP40; R27G01-ZpGdbd in attP2*
driver	*VT019802-LexA*	*VT019802-LexA in attp40* [3]
driver	*VT033282-GAL4*	*VT033282-GAL4 in attp2* [3]
driver	*VT036875-GAL4*	*VT036875-GAL4 in attp2* [3]
driver	*VT036875-LexA*	*VT036875-LexA in attp2* [3]
driver	*VT045584-LexA*	*VT045584-LexA in attp40* [3]

Supplementary Table 1 | Fly stocks.

Figure	Short genotype	Full genotype
1a	vFBs-GAL4> UAS-Lola	+/Y; UAS-FLP.PEST/+; lola>>luc/VT036875-GAL4 in attp2
1b	vFBs-GAL4> UAS-Lola	+/Y; UAS-FLP.PEST/+; lola>>luc/VT036875-GAL4 in attp2
1c	wt-Canton-S	+/Y; +; + (Canton-S)
1d	wt-Canton-S	+/Y; +; + (Canton-S)
1e	wt-Canton-S	+/Y; +; + (Canton-S)
1f	vFBs-GAL4/UAS-Shi^{ts}	+/Y; UAS-Shi^{ts}/R58E02-LexA; VT036875-GAL4/LexAop-GAL80
1f	vFBs-GAL4/WT-cs	+/Y; R58E02-LexA/+; VT036875-GAL4/+
1f	UAS-Shi^{ts}/WT-cs	+/Y; UAS-Shi^{ts}/+; LexAop-GAL80/+
2a	MB002B	UAS-csChrimson/Y; MB002B-GAL4p65adz/+; MB002B-ZpGAL4dbd/+
2a	MB018B	UAS-csChrimson/Y; MB018B-GAL4p65adz/+; MB018B-ZpGAL4dbd/+
2a	MB027B	UAS-csChrimson/Y; MB027B-GAL4p65adz/+; MB027B-ZpGAL4dbd/+
2a	MB051B	UAS-csChrimson/Y; MB051B-GAL4p65adz/+; MB051B-ZpGAL4dbd/+
2a	MB052B	UAS-csChrimson/Y; MB052B-GAL4p65adz/+; MB052B-ZpGAL4dbd/+
2a	MB057B	UAS-csChrimson/Y; MB057B-GAL4p65adz/+; MB057B-ZpGAL4dbd/+
2a	MB077B	UAS-csChrimson/Y; MB077B-GAL4p65adz/+; MB077B-ZpGAL4dbd/+
2a	MB082C	UAS-csChrimson/Y; +/+; MB082C-GAL4p65adz, MB082C-ZpGAL4dbd/+
2a	MB083C	UAS-csChrimson/Y; +/+; MB083C-GAL4p65adz, MB083C-ZpGAL4dbd/+
2a	MB110C	UAS-csChrimson/Y; MB110B-GAL4p65adz/+; MB110B-ZpGAL4dbd/+
2a	MB112C	UAS-csChrimson/Y; MB112B-GAL4p65adz/+; MB112B-ZpGAL4dbd/+
2a	MB242A	UAS-csChrimson/Y; MB242A-GAL4p65adz/+; MB242A-ZpGAL4dbd/+
2a	MB298B	UAS-csChrimson/Y; MB298B-GAL4p65adz/+; MB298B-ZpGAL4dbd/+
2a	MB310C	UAS-csChrimson/Y; MB310B-GAL4p65adz/+; MB310B-ZpGAL4dbd/+
2a	MB399B	UAS-csChrimson/Y; MB399B-GAL4p65adz/+; MB399B-ZpGAL4dbd/+
2a	MB433B	UAS-csChrimson/Y; MB433B-GAL4p65adz/+; MB433B-ZpGAL4dbd/+
2a	MB434B	UAS-csChrimson/Y; MB434B-GAL4p65adz/+; MB434B-ZpGAL4dbd/+
2a	MB542B	UAS-csChrimson/Y; MB542B-GAL4p65adz/+; MB542B-ZpGAL4dbd/+
2a	MB543B	UAS-csChrimson/Y; MB543B-GAL4p65adz/+; MB543B-ZpGAL4dbd/+
2a	MB549C	UAS-csChrimson/Y; +/+; MB549C-GAL4p65adz, MB549C-ZpGAL4dbd/+
2a	MB622B	UAS-csChrimson/Y; MB622B-GAL4p65adz/+; MB622B-ZpGAL4dbd/+
2a	SS01308	UAS-csChrimson/Y; SS01308-GAL4p65adz/+; SS01308-ZpGAL4dbd/+
2a	VT036875-GAL4	UAS-csChrimson/Y; +/+; VT036875-GAL4/+
2a	R23E10-GAL4	UAS-csChrimson/Y; +/+; R23E10-GAL4/+
2a	pBDP	UAS-csChrimson/Y; +/+; pBDP/+
2c	MBON-$\gamma 2\alpha$'1-GAL4>UAS-Shi^{ts}	+/Y; MB077B-GAL4p65adz/UAS-Shi^{ts}; MB077B-ZpGAL4dbd/+
2c	pBDP-GAL4>UAS-Shi^{ts}	+/Y; UAS-Shi^{ts}/+; pBDP-GAL4/+
2d	MBON-$\gamma 2\alpha$'1-GAL4>UAS-Shi^{ts}	+/Y; MB077B-GAL4p65adz/UAS-Shi^{ts}; MB077B-ZpGAL4dbd/+
2d	pBDP-GAL4>UAS-Shi^{ts}	+/Y; UAS-Shi^{ts}/+; pBDP-GAL4/+
2e	MBON-$\gamma 2\alpha$'1-GAL4>UAS-Chrimson88<br>vFBs-LexA>LexAop-GCaMP6s	UAS-Chrimson88, LexAoCp-GCaMP6s/Y; MB077B-GAL4p65adz/+; MB077B-ZpGAL4dbd/VT036875-LexA
2e	MBON-calyx-GAL4>UAS-Chrimson88<br>vFBs-LexA>LexAop-GCaMP6s	UAS-Chrimson88, LexAoCp-GCaMP6s/Y; MB242A-GAL4p65adz/+; MB242A-ZpGAL4dbd/VT036875-LexA
2e	MBON-$\gamma 2\alpha$'1-GAL4>UAS-Chrimson88<br>dFBs-LexA>LexAop-GCaMP6s	UAS-Chrimson88, LexAoCp-GCaMP6s/Y; MB077B-GAL4p65adz/+; MB077B-ZpGAL4dbd/R23E10-LexA
2f	MBON-$\gamma 2\alpha$'1-GAL4/UAS-Lola	+/Y; UAS-FLP.PEST/ MB077B-GAL4p65adz; lola>>luc/MB077B-ZpGAL4dbd
2g	MBON-$\gamma 2\alpha$'1-GAL4/UAS-Lola	+/Y; UAS-FLP.PEST/ MB077B-GAL4p65adz; lola>>luc/MB077B-ZpGAL4dbd
3b	MBON–β'2mp-GAL4>UAS-Chrimson88<br>vFBs-LexA>LexAop-GCaMP6s	UAS-Chrimson88, LexAoCp-GCaMP6s/Y; MB002B-GAL4p65adz/+; MB002B-ZpGAL4dbd/VT036875-LexA
3b	+/UAS-Chrimson88<br>vFBs-LexA>LexAop-GCaMP6s	UAS-Chrimson88, LexAoCp-GCaMP6s/Y; +/+; +/VT036875-LexA
3c	MBON–β'2mp-GAL4>UAS-Lola	+/Y; UAS-FLP.PEST/ MB002B-GAL4p65adz; lola>>luc/MB002B-ZpGAL4dbd
3d	MBON–β'2mp-GAL4>UAS-Lola	+/Y; UAS-FLP.PEST/ MB002B-GAL4p65adz; lola>>luc/MB002B-ZpGAL4dbd
3e	MBON–β'2mp-GAL4>UAS-Shi^{ts}	+/Y; MB002B-GAL4p65adz/UAS-Shi^{ts}; MB002B-ZpGAL4dbd/+
3e	pBDP-GAL4>UAS-Shi^{ts}	+/Y; UAS-Shi^{ts}/+; pBDP-GAL4/+
3f	MBON–β'2mp-GAL4>UAS-Shi^{ts}	+/Y; MB002B-GAL4p65adz/UAS-Shi^{ts}; MB002B-ZpGAL4dbd/+
3f	pBDP-GAL4>UAS-Shi^{ts}	+/Y; UAS-Shi^{ts}/+; pBDP-GAL4/+
3g	MBON–β'2mp-GAL4>UAS-Shi^{ts}	+/Y; MB002B-GAL4p65adz/UAS-Shi^{ts}; MB002B-ZpGAL4dbd/+
3g	pBDP-GAL4>UAS-Shi^{ts}	+/Y; UAS-Shi^{ts}/+; pBDP-GAL4/+
3h	MBON–β'2mp-GAL4>UAS-csChrimson	UAS-csChrimson/Y; MB002B-GAL4p65adz/+; MB002B-ZpGAL4dbd/+
3h	pBDP-GAL4>UAS-csChrimson	UAS-csChrimson /Y; +/+; pBDP-GAL4/+
3i	MBON–β'2mp-GAL4>UAS-csChrimson	UAS-csChrimson/Y; MB002B-GAL4p65adz/+; MB002B-ZpGAL4dbd/+
3i	pBDP-GAL4>UAS-csChrimson	UAS-csChrimson /Y; +/+; pBDP-GAL4/+
4b	VT033282-GAL4>UAS-myrGFP	+/Y; +/+; VT033282-GAL4/UAS-myr::smGFP
4c	MBON-$\gamma 2\alpha$'1-LexA>LexAop-Chrimson88<br>SFSs-GAL4>UAS-GCaMP6s	LexAoCp-Chrimson88, UAS-GCaMP6s/Y; VT019802-LexA/+; VT033282-GAL4/+
4d	MBON–β'2mp-LexA>LexAop-Chrimson88<br>SFSs-GAL4>UAS-GCaMP6s	LexAoCp-Chrimson88, UAS-GCaMP6s/Y; VT045584-LexA/+; VT033282-GAL4/+
4e	SFSs-GAL4>UAS-Chrimson88<br>vFBs-LexA>LexAop-GCaMP6s	UAS-Chrimson88, LexAop-GCaMP6s/Y; +/+; VT033282-GAL4/VT036875-LexA
4f	SFSs-GAL4>UAS-Shi^{ts}	+/Y; UAS-Shi^{ts}/+; VT033282-GAL4/+
4f	pBDP-GAL4> UAS-Shi^{ts}	+/Y; UAS-Shi^{ts}/+; pBDP-GAL4/+
4g	SFSs-GAL4>UAS-Shi^{ts}	+/Y; UAS-Shi^{ts}/+; VT033282-GAL4/+
4g	pBDP-GAL4> UAS-Shi^{ts}	+/Y; UAS-Shi^{ts}/+; pBDP-GAL4/+
4h	SFSs-GAL4>UAS-GCAMP7b	+/Y; SFSs-GCAMP7b/+; VT033282-GAL4/+
4i	SFSs-GAL4>UAS-GCAMP7b	+/Y; UAS-GCAMP7b/+; VT033282-GAL4/+
Suppl. 1a	VT036875-GAL4	+/Y; +/+; VT036875-GAL4/UAS-myr::smGFP
Suppl. 1b	R58E02-LexA	+/Y; R58E02-LexA/+; +/13XLexAop2-IVS-myr::smGFP

Suppl. 1c	MB077B-GAL4	+/Y; MB077B-GAL4p65adz/+; MB077B-ZpGAL4dbd/UAS-myr::smGFP
Suppl. 1d	VT036875-LexA	+/Y; +/+; VT036875-LexA/13XLexAop2-IVS-myr::smGFP
Suppl. 1e	R23E10-LexA	+/Y; R23E10-LexA/+; +/13XLexAop2-IVS-myr::smGFP
Suppl. 1f	MB242A-GAL4	+/Y; MB242A-GAL4p65adz/+; MB242A-ZpGAL4dbd/UAS-myr::smGFP
Suppl. 1g	R23E10-GAL4	+/Y; +/+; R23E10-GAL4/UAS-myr::smGFP
Suppl. 1h	MB002B-GAL4	+/Y; MB002B-GAL4p65adz/+; MB002B-ZpGAL4dbd/UAS-myr::smGFP
Suppl. 1i	VT033282-GAL4	+/Y; +/+; VT033282-GAL4/UAS-myr::smGFP
Suppl. 1j	VT019018-LexA	+/Y; VT019018-LexA/+; +/13XLexAop2-IVS-myr::smGFP
Suppl. 1k	VT045584-LexA	+/Y; VT045584-LexA/+; +/13XLexAop2-IVS-myr::smGFP
Suppl. 2a	dFBs-GAL4>UAS-Lola	+/Y; UAS-FLP.PEST/+; lola>>luc/R23E10-GAL4
Suppl. 2b	vFBs-GAL4>UAS-GCaMP7b	+/Y; UAS-GCAMP7b/+; VT036875-GAL4/+
Suppl. 2c	vFBs-GAL4>UAS-GCAMP7b	+/Y; UAS-GCAMP7b/+; VT036875-GAL4/+
Suppl. 2d	dFBs-GAL4>UAS-GCAMP6s	+/Y; UAS-GCAMP6s/+; R23E10-GAL4/+
Suppl. 2e	DANs−β'1-GAL4>UAS-GCAMP7b	+/Y; UAS-GCAMP7b/+; VT036875-GAL4/+
Suppl. 2f	vFBs	+/Y; UAS-GCAMP7b/+; VT036875-GAL4/+
Suppl. 2f	dFBs	+/Y; UAS-GCAMP6s/+; R23E10-GAL4/+
Suppl. 2f	DANs−β'1	+/Y; UAS-GCAMP7b/+; VT036875-GAL4/+
Suppl. 2g	vFBs-GAL4>UAS-Shi^{ts}	+/Y; UAS-Shi^{ts}/+; VT036875-GAL4/+
Suppl. 2g	pBDP-GAL4> UAS-Shi^{ts}	+/Y; UAS-Shi^{ts}/+; pBDP-GAL4/+
Suppl. 2h	vFBs-GAL4>UAS-Shi^{ts}	+/Y; UAS-Shi^{ts}/+; VT036875-GAL4/+
Suppl. 2h	pBDP-GAL4> UAS-Shi^{ts}	+/Y; UAS-Shi^{ts}/+; pBDP-GAL4/+
Suppl. 2i	wt-Canton-S	+/Y; +; + (Canton-S)
Suppl. 2j	wt-Canton-S	+/Y; +; + (Canton-S)
Suppl. 2k	wt-Canton-S	+/Y; +; + (Canton-S)
Suppl. 2i	wt-Canton-S	+/Y; +; + (Canton-S)
Suppl. 3a	MBON-$\gamma 2\alpha$'1-LexA>LexAop-Shi^{ts}, vFBs-GAL4>UAS-Lola	+/Y; UAS-FLP.PEST/VT019802-LexA; lola>>luc/VT036875-GAL4, LexAop-Shibire^{ts}
Suppl. 3a	+/LexAop-Shi^{ts}, vFBs-GAL4>UAS-Lola	+/Y; UAS-FLP.PEST/+; lola>>luc/VT036875-GAL4, LexAop-Shibire^{ts}
Suppl. 4a	dFBs-GAL4>UAS-Chrimson88<br>vFBs-LexA>LexAop-GCaMP6s	UAS-Chrimson88, LexAop-GCaMP6s/Y; + /+; R23E10-GAL4/VT036875-LexA
Suppl. 4b	vFBs-GAL4>UAS-Chrimson88<br>dFBs-LexA>LexAop-GCaMP6s	UAS-Chrimson88, LexAop-GCaMP6s/Y; R23E10-LexA; +/VT036875-GAL4
Suppl. 4c	SFSs-GAL4>UAS-Chrimson88	UAS-Chrimson88, LexAop-GCaMP6s/Y; +/+; VT033282-GAL4/VT036875-LexA

Supplementary Table 2 | Full genotypes of flies used to generate data presented in each figure.

Figure 1e
wt-Canton-S

Training [hr]	Median CI [%]	n	SI [%]	*P* SI=0	*P* $SI_{2hr}=SI_{4hr}$	Test
0	73.7	58				
1	71.53	60	2.94	0.2942		Permutation test
2	72.81	59	1.21	0.3277	0.0175	Permutation test
4	61.57	63	16.46	0.0022		Permutation test
6	55.87	60	24.2	1.2e-4		Permutation test

Figure 2a

Genotype	Median ΔSleep [min/30min]	n	*P* ΔSleep=0	Test
MB002B	-7.00	54	8.8e-5	Wilcoxon Signed Rank Test
MB018B	-6.25	54	0.0002	Wilcoxon Signed Rank Test
MB027B	0.00	54	0.2225	Wilcoxon Signed Rank Test
MB051B	6.67	36	3.9e-5	Wilcoxon Signed Rank Test
MB052B	-8.83	54	3.2e-8	Wilcoxon Signed Rank Test
MB057B	-1.75	54	0.0048	Wilcoxon Signed Rank Test
MB077B	5.39	36	0.0036	Wilcoxon Signed Rank Test
MB082C	-0.50	54	0.0981	Wilcoxon Signed Rank Test
MB083C	0.00	54	0.3758	Wilcoxon Signed Rank Test
MB110C	-1.88	36	0.1161	Wilcoxon Signed Rank Test
MB112C	-6.08	54	7.1e-7	Wilcoxon Signed Rank Test
MB242A	0.00	54	0.0205	Wilcoxon Signed Rank Test
MB298B	-3.96	54	0.0013	Wilcoxon Signed Rank Test
MB310B	0.00	54	0.3758	Wilcoxon Signed Rank Test
MB399B	-5.50	54	5.8e-6	Wilcoxon Signed Rank Test
MB433B	-2.67	54	0.0244	Wilcoxon Signed Rank Test
MB434B	-5.75	54	0.0001	Wilcoxon Signed Rank Test
MB542B	0.75	54	0.9211	Wilcoxon Signed Rank Test
MB543B	-3.29	54	0.0003	Wilcoxon Signed Rank Test
MB549C	-5.21	54	0.0053	Wilcoxon Signed Rank Test
MB622B	-1.00	54	0.1524	Wilcoxon Signed Rank Test
SS01308	2.29	36	0.0648	Wilcoxon Signed Rank Test
VT036875-GAL4	12.5	36	5.7e-6	Wilcoxon Signed Rank Test
R23E10-GAL4	4.04	36	2.6e-5	Wilcoxon Signed Rank Test
pBDP	0.00	36	0.7051	Wilcoxon Signed Rank Test

Figure 2c

Temp [°C]	Genotype	n	CI [%]	SI [%]	P SI=0	P $SI_{exp}=SI_{ctrl}$	Test
30	*MBON-γ2α'1-GAL4/UAS-Shi^{ts}*	53	52.55				
		51	58.72	-11.74	0.8602		Permutation test
20	*MBON-γ2α'1-GAL4/UAS-Shi^{ts}*	53	54.09				
		54	38.54	28.75	0.0003	0.0105	Permutation test
30	pBDP-*GAL4/UAS-Shi^{ts}*	44	72.74				
		44	52.09	28.39	0.0000	0.0013	Permutation test

Figure 2e

Genotype	Peak ΔF/F [%]	SEM	n	P ΔF/F=0	Test
MBON-γ2α'1-GAL4>UAS-Chrimson88<br>*vFBs-LexA>LexAop-GCaMP6s*	126.28	13.77	7	1.0e-5	Student T-test
MBON-calyx-GAL4>UAS-Chrimson88<br>*vFBs-LexA>LexAop-GCaMP6s*	1.35	2.48	4	0.6254	Student T-test
MBON-γ2α'1-GAL4>UAS-Chrimson88<br>*dFBs-LexA>LexAop-GCaMP6s*	-1.11	1.48	7	0.4812	Student T-test

Figure 3b

genotype	peak ΔF/F [%]	SEM	n	P ΔF/F=0	Test
MBON−β'2mp-GAL4>UAS-Chrimson88<br>*vFBs-LexA>LexAop-GCaMP6s*	-20.8300	6.3400	5	0.0303	Student T-test
+/UAS-Chrimson88<br>*vFBs-LexA>LexAop-GCaMP6s*	5.3700	1.0600	5	0.0071	Student T-test

Figure 3g

Temp [°C]	Genotype	CI [%]	n	SI [%]	P SI=0	P $SI_{20°C}=SI_{31°C}$	Test
20	*MBON−β'2mp-GAL4/UAS-Shi^{ts}*	82.12	53				
		80.30	52	2.22	0.2188		Permutation test
31	*MBON−β'2mp-GAL4/UAS-Shi^{ts}*	71.85	59				
		55.42	61	23.17	0.0019	0.0376	Permutation test
20	pBDP-*GAL4/ UAS-Shi^{ts}*	75.07	52				
		73.96	58	1.47	0.4019		Permutation test
31	pBDP-*GAL4/ UAS-Shi^{ts}*	78.77	72				
		76.92	59	2.34	0.2663	0.8575	Permutation test

Figure 3i

CHR light	Genotype	CI [%]	n	SI [%]	P SI=0	P $SI_{L-}=SI_{L+}$	Test
-	*MBON−β'2mp-GAL4>UAS-csChrimson*	82.99	56				
		64.46	57	22.33	1.0e-5		Permutation test
+	*MBON−β'2mp-GAL4> UAS-csChrimson*	77.80	57				
		73.07	56	6.08	0.0517	0.0403	Permutation test
-	*pBDP-GAL4> UAS-csChrimson*	37.471	30				
		24.24	40	35.31	4.8e-4		Permutation test
+	*pBDP-GAL4> UAS-csChrimson*	52.71	33				
		34.49	37	34.57	1.0e-5	0.9542	Permutation test

	Genotype	Peak ΔF/F [%]	SEM	n	P ΔF/F=0	Test
**Figure 4c**	*MBON-γ2α'1-LexA>LexAop-Chrimson88*<br>*SFSs-GAL4>UAS-GCaMP6s*	10.73	4.59	9	0.0480	Student T-test
**Figure 4d**	*MBON−β'2mp-LexA>LexAop-Chrimson88*<br>*SFSs-GAL4>UAS-GCaMP6s*	-14.55	2.31	5	0.0002	Student T-test
**Figure 4e**	*SFSs-GAL4>UAS-Chrimson88*<br>*vFBs-LexA>LexAop-GCaMP6s*	69.45	16.04	6	0.0075	Student T-test

Figure 4g

Temp [°C]	Genotype	CI [%]	n	SI [%]	P SI=0	P $SI_{20°C}=SI_{30°C}$	Test
20	*SFSs-GAL4>UAS-Shi^{ts}*	74.125	52				
		59.47	52	19.82	1.0e-5		Permutation test
30	*SFSs-GAL4>UAS-Shi^{ts}*	67.18	61				
		67.073	64	0.16	0.4976	0.0115	Permutation test
20	*pBDP-GAL4>UAS-Shi^{ts}*	74.815	59				
		64.45	51	13.84	0.0028		Permutation test
30	*pBDP-GAL4>UAS-Shi^{ts}*	74.12	61				
		62.04	63	16.29	2.1e-4	0.7954	Permutation test

Supplementary Figure 2g

Temp [°C]	Genotype	CI [%]	n	SI [%]	P SI=0	P $SI_{20°C}=SI_{30°C}$	Test
20	*vFBs-GAL4>UAS-Shi^{ts}*	77.58	35				
		41.54	35	46.45	1.0e-5		Permutation test
30	*vFBs-GAL4>UAS-Shi^{ts}*	51.97	41				
		22.96	42	55.81	0.1.1e-4	0.4951	Permutation test

20	*pBDP-GAL4>UAS-Shi*^{ts}	74.24	41				
		24.21	40	67.39	1.0e-5		Permutation test
30	*pBDP-GAL4>UAS-Shi*^{ts}	60.85	39				
		20.56	38	66.21	1.0e-5	0.9021	Permutation test

Supplementary Figure 2h

Temp [°C]	Genotype	CI [%]	n	SI [%]	*P* SI=0	*P* SI$_{20°C}$ =SI$_{30°C}$	Test
20	*vFBs-GAL4>UAS-Shi*^{ts}	68.45	69				
		47.76	61	30.22	1.0e-5		Permutation test
30	*vFBs-GAL4>UAS-Shi*^{ts}	67.77	71				
		65.08	69	3.97	0.2505	0.0028	Permutation test
20	*pBDP-GAL4>UAS-Shi*^{ts}	68.41	71				
		36.75	64	46.28	1.0e-5		Permutation test
30	*pBDP-GAL4>UAS-Shi*^{ts}	70.98	69				
		45.56	65	35.81	1.0e-5	0.2582	Permutation test

Supplementary Figure 4a

Genotype	Peak ΔF/F [%]	SEM	n	*P* ΔF/F=0	Test
dFBs-GAL4>UAS-Chrimson88 *vFBs-LexA>LexAop-GCaMP6s*	1.13	1.49	7	0.4777	Student T-test

Supplementary Figure 4b

Genotype	Peak ΔF/F [%]	SEM	n	*P* ΔF/F=0	Test
vFBs-GAL4>UAS-Chrimson88 *dFBs-LexA>LexAop-GCaMP6s*	-3.86	2.03	6	0.1154	Student T-test

All statistical tests are two-sided.

Supplementary Table 3 | Statistical analysis of data presented in each figure.

Supplementary References

1 Aso, Y. *et al.* The neuronal architecture of the mushroom body provides a logic for associative learning. *Elife* **3**, e04577, doi:10.7554/eLife.04577 (2014).

2 Pfeiffer, B. D. *et al.* Tools for neuroanatomy and neurogenetics in Drosophila. *Proc Natl Acad Sci U S A* **105**, 9715-9720, doi:10.1073/pnas.0803697105 (2008).

3 Tirian, L. & Dickson, B. J. *The VT GAL4, LexA, and split-GAL4 driver line collections for targeted expression in the Drosophila nervous system* (Cold Spring Harbor Laboratory, 2017).
