# Supplementary material for: A neural circuit linking learning and sleep in Drosophila long-term memory
Source: Nat Commun. 2022 Feb 1;13:609. doi: 10.1038/s41467-022-28256-1 (PMC8807839; doi:10.1038/s41467-022-28256-1)
Supplement: Supplementary file 3 — Reporting summary [file 41467_2022_28256_MOESM3_ESM.pdf]

nature research

Corresponding author(s): Krystyna Keleman

Last updated by author(s): December 14, 2021

Reporting Summary

Nature Research wishes to improve the reproducibility of the work that we publish. This form provides structure for consistency and transparency in reporting. For further information on Nature Research policies, see Authors & Referees and the Editorial Policy Checklist .

Statistics

For all statistical analyses, confirm that the following items are present in the figure legend, table legend, main text, or Methods section.

n/a	Confirmed	
☐	☒	The exact sample size (*n*) for each experimental group/condition, given as a discrete number and unit of measurement
☐	☒	A statement on whether measurements were taken from distinct samples or whether the same sample was measured repeatedly
☐	☒	The statistical test(s) used AND whether they are one- or two-sided<br>*Only common tests should be described solely by name; describe more complex techniques in the Methods section.*
☒	☐	A description of all covariates tested
☐	☒	A description of any assumptions or corrections, such as tests of normality and adjustment for multiple comparisons
☐	☒	A full description of the statistical parameters including central tendency (e.g. means) or other basic estimates (e.g. regression coefficient) AND variation (e.g. standard deviation) or associated estimates of uncertainty (e.g. confidence intervals)
☐	☒	For null hypothesis testing, the test statistic (e.g. *F*, *t*, *r*) with confidence intervals, effect sizes, degrees of freedom and *P* value noted<br>*Give P values as exact values whenever suitable.*
☒	☐	For Bayesian analysis, information on the choice of priors and Markov chain Monte Carlo settings
☒	☐	For hierarchical and complex designs, identification of the appropriate level for tests and full reporting of outcomes
☒	☐	Estimates of effect sizes (e.g. Cohen's *d*, Pearson's *r*), indicating how they were calculated

Our web collection on statistics for biologists contains articles on many of the points above.

Software and code

Policy information about availability of computer code

Data collection	Bioluminescence data are collected with SparkControlTM (v2.6 TECAN). Videos of behaving flies for courtship or sleep measurement are collected by jVision (Rel F) software by Janelia jET. csChrimson activation on behaving flies were controlled by customized Matlab program RGBCtrl (v3.1). Calcium imaging data are collected with ScanImage 2016 (Virdrio Technologies, LLC) running in Matlab (R2016b MathWorks). The codes used for the data collection are available in the GitHub repository.
Data analysis	Courtship indexes are derived by the automated video analysis software Matebook (Ver 2141). Sleep levels are calculated with the SleepTracker (v1.1, customized Matlab software). Calcium imaging data are analyzed in ImageJ (1.53c, Fiji) and customized Matlab (R2019b MathWorks) code. Neuron expression pattern was extracted from light microscopy stacks in VVD viewer (Ver. 161214). All statistic analysis and data visualizations are done in Matlab (R2019b MathWorks) with customized codes. The codes used for the data analysis and to generate the figures are available in the GitHub repository.

For manuscripts utilizing custom algorithms or software that are central to the research but not yet described in published literature, software must be made available to editors/reviewers. We strongly encourage code deposition in a community repository (e.g. GitHub). See the Nature Research guidelines for submitting code & software for further information.

Data

Policy information about availability of data

All manuscripts must include a data availability statement. This statement should provide the following information, where applicable:

- Accession codes, unique identifiers, or web links for publicly available datasets
- A list of figures that have associated raw data
- A description of any restrictions on data availability

Source data are provided with this manuscript.

Field-specific reporting

Please select the one below that is the best fit for your research. If you are not sure, read the appropriate sections before making your selection.

- [x] Life sciences
- [ ] Behavioural & social sciences
- [ ] Ecological, evolutionary & environmental sciences

For a reference copy of the document with all sections, see nature.com/documents/nr-reporting-summary-flat.pdf

Life sciences study design

All studies must disclose on these points even when the disclosure is negative.

Sample size	Samples sizes were chosen a priori according to typical sample sizes in the field. Ejima A et al. Curr Biol. 2005, Ishimoto H. et al. PNAS, 2009
Data exclusions	No data were excluded.
Replication	Each experiment was performed in 3-5 independent biological replicas. All attempts at replication were successful.
Randomization	Samples were allocated based on the corresponding genotypes.
Blinding	Investigators were not blinded to genotypes during data collection and analysis, as the data collection and analysis were fully automated.

Reporting for specific materials, systems and methods

We require information from authors about some types of materials, experimental systems and methods used in many studies. Here, indicate whether each material, system or method listed is relevant to your study. If you are not sure if a list item applies to your research, read the appropriate section before selecting a response.

Materials & experimental systems

n/a	Involved in the study	
x		Antibodies
x		Eukaryotic cell lines
x		Palaeontology
	x	Animals and other organisms
x		Human research participants
x		Clinical data

Methods

n/a	Involved in the study	
x		ChIP-seq
x		Flow cytometry
x		MRI-based neuroimaging

Animals and other organisms

Policy information about studies involving animals; ARRIVE guidelines recommended for reporting animal research

Laboratory animals	Drosophila melanogaster were used in this study. All experimental animals were males aged 5 to 9 days, depending on the assay. Females, 4 days old, were used as trainers or testers.
Wild animals	This study did not involve wild animals.
Field-collected samples	This study did not involve samples collected from the field.
Ethics oversight	No ethical approval was required.

Note that full information on the approval of the study protocol must also be provided in the manuscript.
